# Suicide Rate Trends for Post–September 11, 2001, US Military Veterans

**DOI:** 10.1001/jamanetworkopen.2025.30216

**Published:** 2025-09-03

**Authors:** Jeffrey T. Howard, Ian J. Stewart, Mary Jo Pugh

**Affiliations:** 1 Department of Public Health, University of Texas at San Antonio, San Antonio; 2 Department of Medicine, Uniformed Services University of Health Sciences, Bethesda, Maryland; 3 Military Cardiovascular Outcomes Research Program, Uniformed Services University of Health Sciences, Bethesda, Maryland; 4 Informatics, Decision-Enhancement, and Analytic Sciences (IDEAS) Center of Innovation, VA Salt Lake City Health Care System, Salt Lake City, Utah; 5 Division of Epidemiology, Department of Internal Medicine, University of Utah School of Medicine, Salt Lake City

## Abstract

This cohort study analyzes data from 2006 through 2022 to investigate suicide rate trends among post–September, 11, 2001 (9/11), US military veterans and the general US adult population.

## Introduction

In 2022, suicide was the 11th leading cause of death in the US, accounting for 49 476 deaths.^1^ Recent studies reported higher suicide rates among post–September 11, 2001 (hereafter, 9/11) military veterans compared with the total US adult population, with rates notably higher for veterans with exposure to traumatic brain injury (TBI), and that rates increased from 2006 to 2020.^2,3^ We analyzed data from 2006 through 2022 to examine whether these trends changed after 2020.

## Methods

This is a retrospective cohort study of military veterans who (1) served on active duty in the US military after 9/11, (2) were aged 18 years or older, and (3) received at least 3 years of care in the Military Health System (MHS).^2^ At least 2 years of care were required for veterans enrolled in the Veterans Health Administration (VHA).^2^ Veteran mortality data were obtained from the National Death Index from 2006 to 2022. Mortality data for the US adult population were obtained from the Centers for Disease Control and Prevention (CDC) WONDER (Wide-Ranging Online Data for Epidemiologic Research)^4^ database for 2006 to 2022. The study was approved by the University of Utah institutional review board, was conducted in accordance with all applicable federal regulations, and followed the STROBE reporting guidelines. Informed consent was not needed because the data were deidentified, aggregated data, in accordance with 45 CFR §46.

Age group (18-24, 25-34, 35-44, 45-54, 55-64, and ≥65 years) and sex (male or female) were obtained from MHS, VHA, and CDC administrative databases with aggregate population and death counts for age and sex standardization. TBI was identified by positive screening on the Comprehensive TBI Evaluation protocol or medical diagnosis of mild, moderate, severe, or penetrating TBI. Suicide death was determined from *International Statistical Classification of Diseases and Related Health Problems, Tenth Revision,* diagnosis codes X60 to X84.

Negative binomial regression models were used to estimate period-specific age-standardized and sex-standardized rates reported as suicide rates with 95% CIs. Change-point regression was used to assess whether trends in suicide rates changed from 2006 to 2022 (segmented package).^5^ Results are reported as rates per 100 000 person-years, annual percentage change (APC), 95% CIs and 2-sided *P*-values, with significance set at *P* < .05. Analyses were conducted from April 15, 2024, through May 10, 2025. Analyses were performed in R version 4.4.2 (R Foundation for Statistical Computing).

## Results

There were a total of 2 530 847 veterans (23 096 066 person-years) with 9667 suicides; the median (IQR) age at cohort entry was 30 (24-40) years, and the median (IQR) follow-up time was 10 (7-13) years. Veteran suicide rates increased from 2006 to 2020 and then decreased from 2020 to 2022 (Figure). Two change-points were detected for each group (Table). Suicide rates for veterans with TBI increased from 2006 to 2011 (APC, 7.66; 95% CI, 4.78 to 1.54; *P* < .001), increased at a slower percentage rate annually from 2011 to 2020 (APC, 5.59; 95% CI, 4.41 to 6.76; *P* < .001), and then decreased from 2020 to 2022 (APC, −12.10; 95% CI, −18.54 to −5.66; *P* = .002) (Table). For veterans without TBI, rates increased from 2006 to 2011 (APC, 4.85; 95% CI, 2.98 to 6.72; *P* < .001), increased at a slower rate from 2011 to 2020 (APC, 3.41; 95% CI, 2.76 to 4.06; *P* < .001); the change in rates from 2020 to 2022 was not statistically significant (APC, −7.60; 95% CI, −15.94 to 0.75; *P* = .07) (Table). The US adult population suicide rates showed a different pattern, increasing from 2006 to 2018 (APC, 0.38; 95% CI, 0.35 to 0.40; *P* < .001), decreasing from 2018 to 2020 (APC, −0.64; 95% CI, −0.85 to −0.43; *P* < .001), and increasing again from 2020 to 2022 (APC, 0.52; 95% CI, 0.10 to 0.94; *P* = .02).

**Figure.**
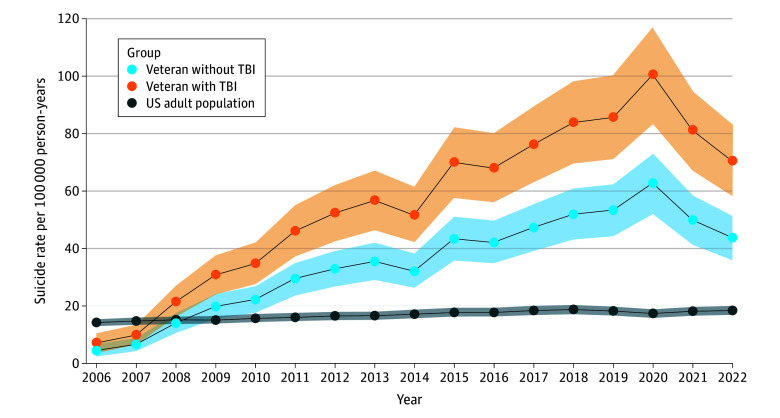
Age- and Sex-Standardized Suicide Rates From 2006 to 2022 Shaded areas around lines denote 95% CIs. TBI indicates traumatic brain injury.

**Table.** Results of Change-Point Regression Analysis of Suicide Rate Trends From 2006 to 2022

Cohort and segment	APC (95% CI)	*P* value
**Veteran with TBI (2 change-points)**		
Segment 1: 2006-2011	7.66 (4.78 to 10.54)	<.001
Segment 2: 2011-2020	5.59 (4.41 to 6.76)	<.001
Segment 3: 2020-2022	−12.10 (−18.54 to −5.66)	.002
**Veteran without TBI (2 change-points)**		
Segment 1: 2006-2011	4.85 (2.98 to 6.72)	<.001
Segment 2: 2011-2020	3.41 (2.76 to 4.06)	<.001
Segment 3: 2020-2022	−7.60 (−15.94 to 0.75)	.07
**US adult population (2 change-points)**		
Segment 1: 2006-2018	0.38 (0.35 to 0.40)	<.001
Segment 2: 2018-2020	−0.64 (−0.85 to −0.43)	<.001
Segment 3: 2020-2022	0.52 (0.10 to 0.94)	.02

Abbreviations: APC, annual percentage change; TBI, traumatic brain injury.

## Discussion

This cohort study found that, after increasing from 2006 to 2020 for veterans with and without TBI,^3^ veteran suicide rates declined from 2020 to 2022. Government programs, such as the Prevention 2.0 Initiative, the Suicide Prevention Now initiative, or the President’s Roadmap to Empower Veterans, may be contributing to reductions in suicide. Evaluation of these potential impacts is critical as the government considers budget cuts to VA programs. Limitations include potential misclassification of causes of death, underreporting of TBI exposure, exclusion of veterans not seeking care in the MHS or VHA, and residual confounding from differences between the veteran and US adult population.

## Notes

Supplement.
Data Sharing Statement
