# Supplementary material for: Suicide Rate Trends for Post–September 11, 2001, US Military Veterans
Source: JAMA Netw Open. 2025 Sep 3;8(9):e2530216. doi: 10.1001/jamanetworkopen.2025.30216 (PMC12558250; doi:10.1001/jamanetworkopen.2025.30216)
Supplement: Supplement. — Data Sharing Statement [file jamanetwopen-e2530216-s001.pdf]

Data Sharing Statement

Howard. Suicide Rate Trends for Post–September 11, 2001, US Military Veterans. *JAMA Netw Open*. Published September 03, 2025. doi:10.1001/jamanetworkopen.2025.30216

Data
Data available: No

Additional Information
Explanation for why data not available: The authors do not control access to the data, therefore we cannot offer to share data. The data are owned by the VA and DoD and require permissions to access. We would however be willing to advise others on how to go through the process of acquiring these permissions.
